# Use of iliac screw associated with more correction of lumbar lordosis than S2-alar-iliac screw for adult spinal deformity

**DOI:** 10.1186/s12891-021-04568-z

**Published:** 2021-08-10

**Authors:** Qiang Luo, Yong-Chan Kim, Ki-Tack Kim, Kee-Yong Ha, Joonghyun Ahn, Sung-Min Kim, Min-Gyu Kim

**Affiliations:** 1 Department of Orthopaedic Surgery, College of Medicine, Kyung Hee University Hospital at Gangdong, Kyung Hee University, 892 Dongnam-ro, Gangdong-gu, Seoul, 05278 South Korea; 2 Department of Orthopaedic Surgery, Graduate School of Medicine, Kyung Hee University, Kyungheedae-ro, Dongdaemun-gu, Seoul, South Korea

**Keywords:** Adult spinal deformity, Iliac screw, s2-alar-iliac screw, Spinopelvic parameters

## Abstract

**Background:**

To date, there is a paucity of reports clarifying the change of spinopelvic parameters in patients with adult spinal deformity (ASD) who underwent long segment spinal fusion using iliac screw (IS) and S2-alar-iliac screw (S2AI) fixation.

**Methods:**

A retrospective review of consecutive patients who underwent deformity correction surgery for ASD between 2013 and 2017 was performed. Patients were divided into two groups based on whether IS or S2AI fixation was performed. All radiographic parameters were measured preoperatively, immediately postoperatively, and the last follow-up. Demographics, intraoperative and clinical data were analyzed between the two groups. Additionally, the cohort was subdivided according to the postoperative change in pelvic incidence (PI): subgroup (C) was defined as change in PI ≥5° and subgroup (NC) with change < 5°. In subgroup analyses, the 2 different types of postoperative change of PI were directly compared.

**Results:**

A total of 142 patients met inclusion criteria: 111 who received IS and 31 received S2AI fixation. The IS group (65.6 ± 26°, 39.8 ± 13.8°) showed a significantly higher change in lumbar lordosis (LL) and upper lumbar lordosis (ULL) than the S2AI group (54.4 ± 17.9°, 30.3 ± 9.9°) (*p* < 0.05). In subgroup (C), PI significantly increased from 53° preoperatively to 59° postoperatively at least 50% of IS cohort, with a mean change of 5.8° (*p* < 0.05). The clinical outcomes at the last follow-up were significantly better in IS group than in S2AI group in terms of VAS scores for back and leg. The occurrence of sacroiliac joint pain and pelvic screw fracture were significantly greater in S2AI group than in IS group (25.8% vs 9%, *p* < 0.05) and (16.1% vs 3.6%, p < 0.05).

**Conclusions:**

Compared with the S2AI technique, the IS technique usable larger cantilever force demonstrated more correction of lumbar lordosis, and possible increase in pelvic incidence. Further study is warranted to clarify the clinical impaction of these results.

## Background

Spinopelvic fixation (SPF) is becoming an increasingly important avenue for degenerative conditions as the aging population grows. This instrumentation technique can also be applied in high-grade spondylolisthesis, trauma, tumors or infection. However, achieving solid fixation in lumbosacral junction continues to be a challenge for spine surgeons because of the tremendous biomechanical forces demand across the junctional area, complex regional anatomy, and a high pseudarthrosis rate, especially in patients with adult spinal deformity (ASD) [1–5]. With the emergence of advanced spinal instrumentation, multiple options have been described for additional SPF over the past decades. Currently, iliac screw (IS) and S2-alar-iliac (S2AI) screw fixations are the most popular method of SPF in clinical practice.

The IS had a long-established history of improving stability, with comparative advantages of greater diameter and length bolts could be used, higher pullout strength, and easier application [6]. However, there are some disadvantages regarding the IS, including the need for lateral connector, more extensive tissue dissection, prominent hardware, and wound dehiscence etc. [3, 7, 8]. Tsuchiya et al. [9] reported that up to 34% ASD patients treated via IS technique necessitating reoperations due to prominence.

In response to above drawbacks, the S2AI has recently become an increasingly popular technique as an alternative method of SPF, which was initially described by Dr. Sponseller [10]. This technique prevented hardware prominence owing to its deeper and more medial entry point compared to IS [7]. Additional, current literature reported the S2AI had several potential advantages over IS with lower rates of reoperation, wound dehiscence, and implant failure [4, 11–13]. However, there are limited data regarding comparative data between these techniques. To date, few studies exist specifically focused on the radiographic change of spinopelvic parameters after SPF utilizing IS and S2AI techniques. Therefore, the aim of this study was to compare the radiographic change of sagittal spinopelvic parameters between these two techniques for the treatment of ASD, and assess the complication rate of our substantial case series.

## Methods

After institutional review board approval, we performed a retrospective review of consecutive patients who underwent long-segment (≥6 levels) spinal fusion and pelvic fixation using IS or S2AI technique at a single institution between 2013 and 2017. The indications for surgery included frequent recurrent low-back and/or leg pain, neurological deficits, severe disability and/or progressive deformity that failed to better with conservative treatment for more than 6 months. We included ASD patients with age ≥ 60 years and at least one of the following: C7 sagittal vertical axis (SVA) > 50 mm, pelvic tilt (PT) > 25°, pelvic incidence (PI) – lumbar lordosis (LL) > 10°. Patients with a history of neuromuscular diseases, malignancy, infection, and postoperative follow-up less than 24 months were excluded. Furthermore, patients were also excluded from this study if they had ankylosing spondylitis, Parkinson’s disease, or incomplete radiographic and clinical records. Eligible patients were divided into 2 groups according to the surgical method: IS group (iliac screw fixation) and S2AI group (S2-alar-iliac screw fixation).

The clinical data for this current cohort were obtained from the electronic medical records and operative database of our institution. Standard demographic data (e.g., age, gender, primary diagnosis, medical comorbidities, and American Society of Anesthesiologists [ASA] [14] classification) were extracted. Surgical parameters included the operated levels, estimated blood loss (EBL), surgical duration, use of an osteotomy, and length of stay (LOS). The major complications requiring revision included proximal junctional kyphosis (PJK), pseudarthrosis, deep infection, sacroiliac joint (SIJ) pain, or other implant-related complications (pelvic screw loosening, fracture, and wound dehiscence etc.) were compared. Clinical results were evaluated using the Visual Analog Scale (VAS) [15] of back and leg pain and Oswestry Disability Index (ODI) [16] at preoperative and 3 months postoperative, and the last follow-up.

### Surgical procedures

**Fig. 1:**
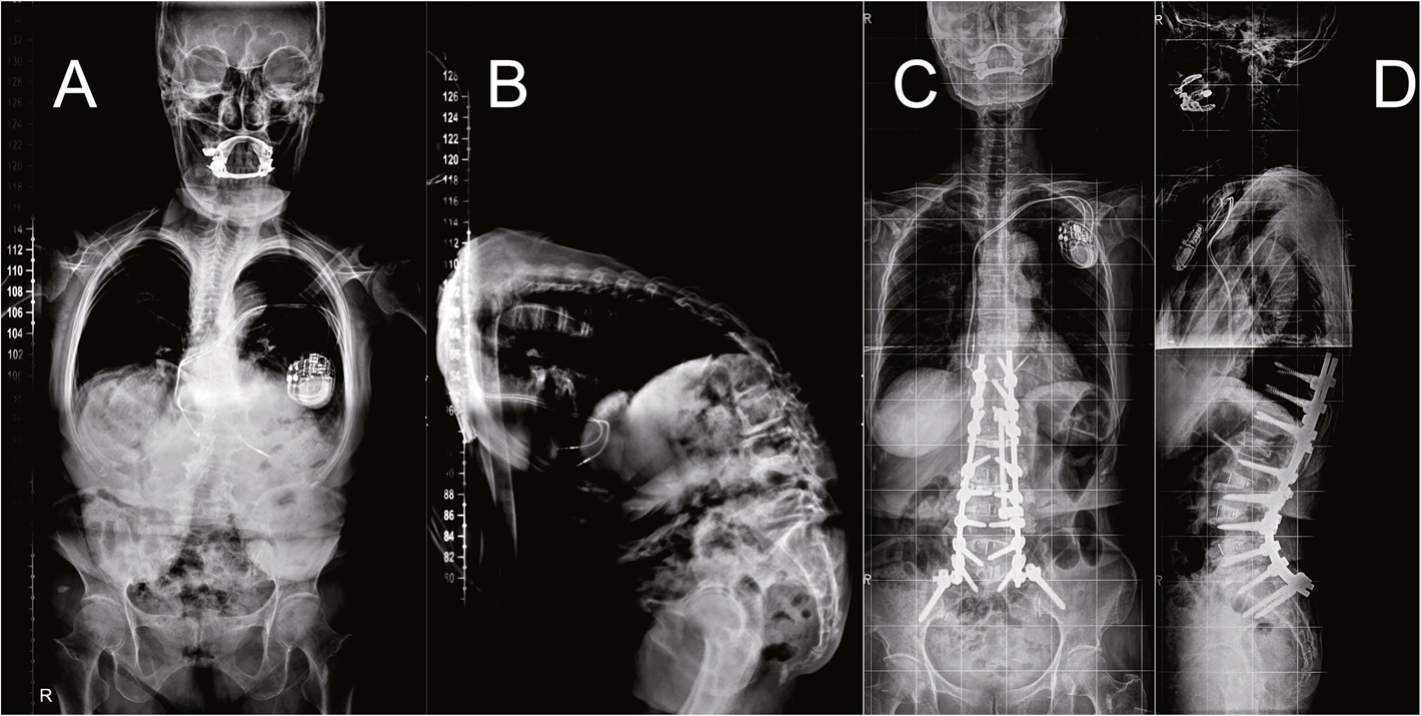
A 78-year-old female patient from IS group. The SVA, PT, and LL were 337.7 mm, 40.4° and 36.4° preoperatively (**A** and **B**) and changed to 12 mm, 18.5°, and − 49.9° after surgery (**C** and **D**). IS indicates iliac screw

The IS procedure was performed base on the technique described previously [1, 6, 17–19]. With patients in the prone position, suprafascial dissection was performed via a midline incision. Routine exposure of the spinous process, lamina, facet joints, and transverse process at the index levels. The bilateral posterior superior iliac spine (PSIS) were then identified. Adequate subperiosteal dissection was performed and splitted fascia longitudinally over the PSIS midline, then stripped to the sides for fascial integrity. The ideal entry point of IS was about 10 mm between the posterior edge of the iliac crest and posterior sacrum. An osteotome was used to remove a small tri- cortical wedge around this point. A straight probe was gently and cautiously inserted into the cancellous channel of ilium, avoiding penetration of inner and outer cortex. Next, the trajectory was tapped with a ball-tip probe to verify the integrity of cortical bone. Then the screw was deeply inserted toward PSIS until its head was flush with the cortex of the ilium to lower the risk of prominence. Finally, the screw was attached to the rod underneath S1 pedicle screw using a lateral connector (Fig. 1).

**Fig. 2:**
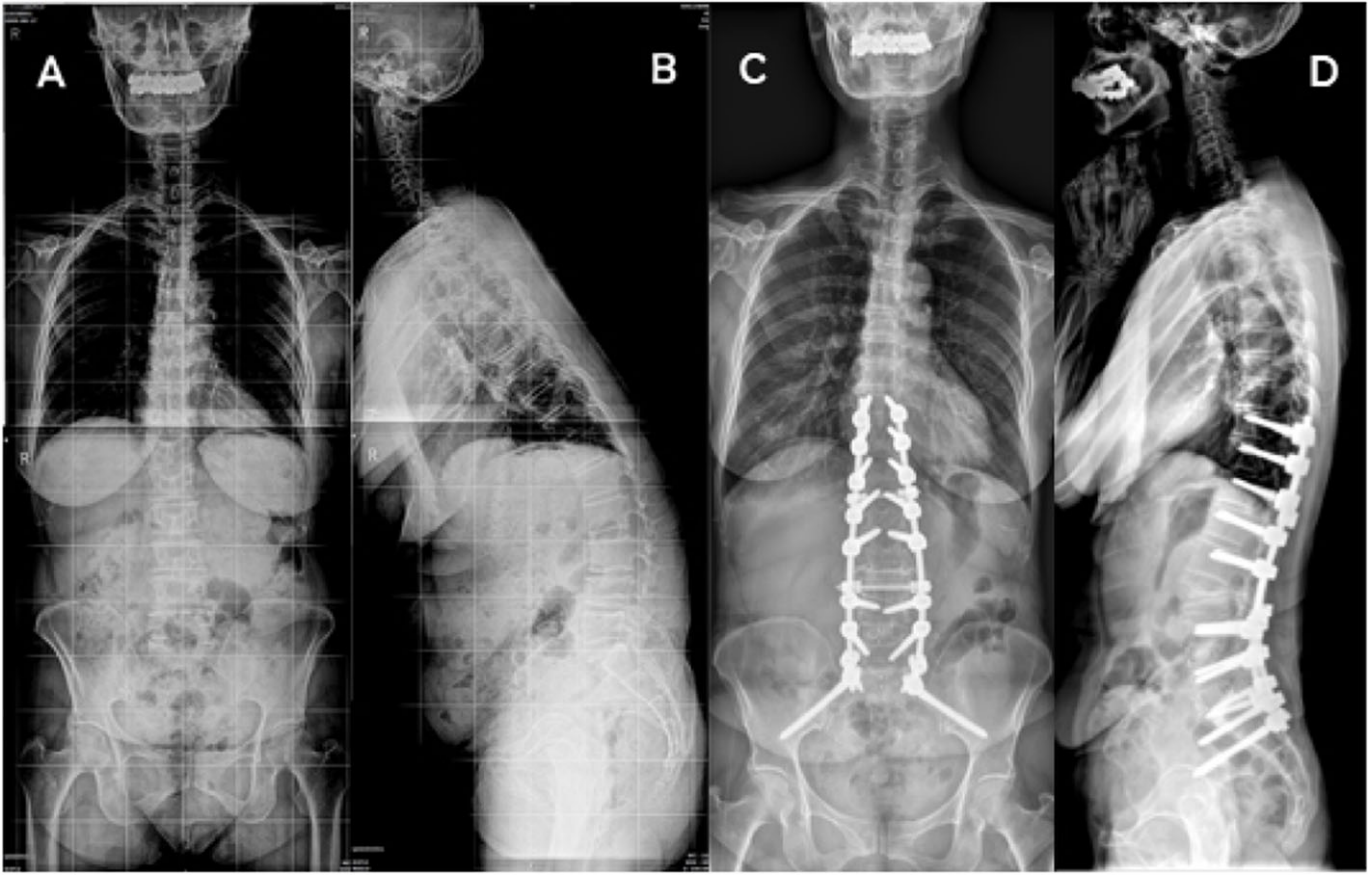
A 72-year-old female patient from S2AI group. The SVA, PT, and LL were 200.1 mm, 27.6° and 2.5° preoperatively (**A** and **B**) and changed to 9.2 mm, 17.6°, and − 58.5 after surgery (**C** and **D**). S2AI indicates S2-Alar-iliac

The procedure of S2AI fixation was performed using a free hand technique described previously [7, 20–24]. A routine posterior exposure was performed similar as IS. S2AI technique has a sacral ala entry point locating distal to the S1 foramen with the angulation of trajectory approximately 30–45° laterally and 25–40° distally, aiming toward the greater trochanter of the femur. Using fluoroscopy to ensure position cephalad to the greater sciatic notch and tap drilling until the SIJ, then reverse drilling until the iliac cortex was reached. Finally, the screw was inserted until its head placed in-line with S1 pedicle screw head (Fig. 2).

We commonly used the pelvic screw with a diameter of 8.5 mm and a length of 70 mm. All cases were performed as 2-stage anteroposterior fusion with an interval of 1 week by the two senior authors (KKT and KYC) as a team with > 20 years of experience in ASD. The IS and S2AI techniques were equally selected from May 2013 to December 2015, but IS was preferentially utilized from January 2016, since we were aware of the advantage of IS technique for getting cantilever force.

### Radiological evaluation

All radiological analysis was performed on lateral radiographs of whole spine (36-in.) obtained preoperatively, postoperatively (first erect) and at the last follow-up according to an established positioning protocol [25]. Spinopelvic parameters included in this analysis were SVA, PT, PI, LL (L1–S1), TK (thoracic kyphosis: T5–12), ULL (upper lumbar lordosis: L1–4), and LLL (lower lumbar lordosis: L4-S1) [26]. Kyphosis was indicated by a (+) value whereas lordosis was indicated by a (−) value. Change of parameter was calculated by subtracting the preoperative value from the postoperative value. All the parameters were measured by two surgeons who did not participate in the operation and the mean value were adopted.

Additionally, we defined PJK was considered present when proximal junctional angle was > 10° and at least 10° greater than the preoperative measurement, which was a severe complication after deformity surgery with radiographic evidence of acute PJK deformity and mechanical failure at the upper instrumented vertebra (UIV) [27]. Fusion was confirmed by the presence of bridging bone connecting the adjacent vertebral bodies either through the implants or around the implants or < 3 mm of translation on flexion or extension radiographs [28–30]. Screw loosening was defined as a radiolucent area ≥ 2 mm surrounding the screw on radiographic images, also known as the “double halo sign [31, 32]”. SIJ pain was defined as unilateral buttock pain meeting the following criteria within 3 months of surgery: SIJ score > 4 [33]; no implant misplacement and prominence; and no surgical site dehiscence and infection.

### Statistical analysis

Results were expressed as mean ± SD. We used the Student’s t-test for continuous variables, and the Chi-square test used for categorical variables. All statistical analyses were performed using SPSS 21.0 software (SPSS Inc., Chicago, IL, USA) with *p* values of < 0.05 considered to be statistically significant.

## Results

### Patient characteristics and perioperative data

In total, 142 patients with a mean follow-up duration of 32.3 months were included in the study and analysis: 111 received IS technique and 31 received S2AI technique. The mean age was 67.3 years and the BMI 28.3 kg/m^2^, 82.4% of patients were female. With regard to surgical data, the mean number of levels fused (8.8 vs 8.7, *p* = 0.084) between the 2 groups were not significantly different. The mean estimated blood loss (EBL) were 2421 ml and 2368 ml in IS and S2AI groups, respectively (*p* > 0.05). Mean surgical duration in IS group was slightly more than in S2AI group (388.2 ± 99.1 min vs 377.6 ± 102.1 min, *p* = 0.602). As shown in Table 1, the comparative analyses revealed no significant intergroup differences in age, sex, primary diagnosis, comorbidities, LOS, and other perioperative data.

**Table 1:** Baseline demographics and surgical data between the IS and S2AI groups

	IS	S2AI	*p*
No. of patients	111	31	
Age, (yrs)	67.9 ± 16.7	65.2 ± 15.8	0.422
Female sex, n (%)	92 (82.8)	25 (80.6)	0.772
BMI, (kg/m^2^)	28.5 ± 8.7	27.6 ± 8.2	0.607
Follow-up, (mos)	32.8 ± 8.0	30.7 ± 6.2	0.179
Smoking, n (%)	12 (10.8)	5 (16.1)	0.530
Diabetes, n (%)	32 (28.8)	9 (29.0)	0.982
Osteoporosis, n (%)	53 (47.7)	13 (41.9)	0.566
Hypertension, n (%)	11 (9.9)	5 (16.1)	0.518
Primary diagnosis, n (%)			0.891
Degenerative	72 (64.95)	19 (61.3)	
Idiopathic	14 (12.6)	4 (12.9)	
Congenital	7 (6.3)	3 (9.7)	
Post-traumatic	18 (16.2)	5 (16.1)	
ASA class, n (%)			0.379
I	14 (12.6)	7 (22.6)	
II	79 (71.2)	19 (61.3)	
III	18 (16.2)	5 (16.1)	
No. of levels fused	8.82 ± 3.7	8.65 ± 7.6	0.084
UIV level, n (%)			0.802
Above T10	9 (8.1)	2 (6.5)	
T10	89 (80.2)	24 (77.4)	
Below T10	13 (11.7)	5 (16.1)	
Interbody fusion level			
L1/2	11 (9.9)	2 (6.5)	0.734
L2/3	101 (90.9)	27 (87.1)	0.506
L3/4	109 (98.2)	29 (93.5)	0.208
L4/5	110 (99.1)	30 (96.8)	0.390
L5/S1	109 (98.2)	30 (96.8)	0.525
PSO level, n (%)			
L2	5 (4.5)	2 (6.5)	0.647
L3	8 (7.2)	3 (9.7)	0.705
L4	2 (1.8)	1 (3.2)	0.525
Surgical duration (mins)	388.2 ± 99.1	377.6 ± 102.1	0.602
Blood loss (ml)	2421.2 ± 255.1	2368.3 ± 354.1	0.353
Length of stay (days)	20.6 ± 5.7	19.1 ± 6.8	0.217

*IS* Iliac screw, *S2AI* S2-alar-iliac, *BMI* Body mass index, *ASA* American Society of Anesthesiologists, *UIV* Upper instrumented vertebra, *PSO* Pedicle subtraction osteotomies

### Radiographic outcomes

Changes in the radiological spinopelvic parameters of all patients are summarized in Table 2. At enrollment, there were no differences in any preoperative parameters between the two groups. Except PI, the improvement achieved after surgery were maintained in all parameters, and subsequently maintained at the last follow-up. LL significantly changed from 11.4 ± 23.9° preoperatively to − 54.2 ± 10° postoperatively in IS group, ULL was 7.1 ± 8.4° preoperatively and − 32.7 ± 9.7° after surgery. LL significantly changed from 12.1 ± 5.3° preoperatively to − 42.3 ± 7.8° postoperatively in S2AI group, ULL was 6.6 ± 11.9° preoperatively and − 23.7 ± 9.3° after surgery. In IS group, the mean preoperative SVA of 155.5 mm decreased to 20.9 mm after surgery and was 46.8 mm at the last follow-up. In S2AI group, the mean preoperative SVA of 139.3 mm decreased to 32.2 mm after surgery and was 67.2 mm at the last follow-up. The mean PT was 37.1° preoperatively, improved to 17.4° postoperatively, and changed to 20.3° at the last follow-up in IS group. It was 38.3° before surgery, corrected to 26.2° after surgery, and changed to 27.7° at the last follow-up in S2AI group.

**Table 2:** Comparison of spinopelvic parameters between the IS and S2AI groups

Parameter	IS	S2AI	*p*
SVA (mm)			
Preop	155.5 ± 55.3	139.3 ± 41.9	0.133
Postop	20.9 ± 33.5	32.2 ± 30.4	0.093
Last follow-up	46.8 ± 51.0	67.2 ± 69.0	0.07
Change	− 134.6 ± 49.3	− 107.1 ± 55.3	**0.005**
TK (°)			
Preop	7.1 ± 12.2	8.1 ± 12.0	0.686
Postop	26.9 ± 8.1	24.1 ± 10.9	0.119
Last follow-up	29.6 ± 9.9	27.0 ± 11.2	0.211
Change	19.8 ± 10.3	16.0 ± 12.0	0.082
PT (°)			
Preop	37.1 ± 8.7	38.3 ± 10.1	0.514
Postop	17.4 ± 7.2	26.2 ± 8.5	**< 0.001**
Last follow-up	20.3 ± 7.6	27.7 ± 8.9	**< 0.001**
Change	−19.7 ± 11.9	−12.1 ± 8.9	**< 0.001**
PI (°)			
Preop	54.8 ± 9.5	56.8 ± 11.4	0.324
Postop	56.1 ± 8.4	56.2 ± 10.9	0.956
Last follow-up	55.9 ± 10.6	56.6 ± 9.7	0.741
Change	1.3 ± 4.3	−0.6 ± 7.3	0.069
LL (°)			
Preop	11.4 ± 23.9	12.1 ± 5.3	0.872
Postop	−54.2 ± 10.0	−42.3 ± 7.8	**< 0.001**
Last follow-up	−51.7 ± 13.8	−39.5 ± 8.1	**< 0.001**
Change	−65.6 ± 26.0	− 54.4 ± 17.9	**0.028**
ULL (°)			
Preop	7.1 ± 8.4	6.6 ± 11.9	0.791
Postop	−32.7 ± 9.7	−23.7 ± 9.3	**< 0.001**
Last follow-up	−29.9 ± 8.6	−21.3 ± 10.4	**< 0.001**
Change	− 39.8 ± 13.8	− 30.3 ± 9.9	**< 0.001**
LLL (°)			
Preop	4.3 ± 7.1	5.5 ± 9.9	0.449
Postop	− 21.5 ± 10.4	−18.6 ± 10.8	0.176
Last follow-up	−18.8 ± 9.7	−18.2 ± 9.4	0.760
Change	−25.8 ± 19.9	−24.1 ± 13.5	0.655

*IS* Iliac screw, *S2AI* S2-alar-iliac, *SVA* Sagittal vertical axis, *TK* Thoracic kyphosis, *PT* Pelvic tilt, *PI* Pelvic incidence, *LL* Lumbar lordosis, *ULL* Upper lumbar lordosis, *LLL* Lower lumbar lordosis, *Preop* Preoperative, *Postop* Postoperative; Change, postoperative - preoperative

Intergroup analysis showed that, IS group had significantly greater magnitudes of improvement from preoperative to postoperative in SVA, PT, LL and ULL. Preoperative values for SVA decreased 65.6 ± 26° in IS group vs 39.8 ± 13.8° in S2AI group (*p* < 0.05). The IS group also experienced greater reduction of PT (IS: − 19.7° vs S2AI: − 12.1°; *p* < 0.01). IS group had larger increases in LL (IS: 66° vs S2AI: 54°; *p* = 0.028) and ULL (IS: 40° vs S2AI: 30°; p < 0.01) than S2AI group.

For the further analysis, the cohort was subdivided based on the postoperative change in PI (Table 3): a change ≥5° subgroup (C) (IS = 64, S2AI = 4) and a change < 5° subgroup (NC) (IS = 47, S2AI = 27). In subgroup (C), PI significantly increased from 53.3° preoperatively to 59.1° postoperatively at least 50% of IS cohort, with a mean change of 5.8° (*p* = 0.032).

**Table 3:** Stratification based on the postoperative change in PI between the IS and S2AI groups

	Subgroup (C)				Subgroup (NC)			
	IS 64 (57.7%)	*p*	S2AI 4 (12.9%)	*p*	IS 47 (42.3%)	*p*	S2AI 27 (87.1%)	*p*
Preop	53.3 ± 14.1		57.9 ± 10.8		56.3 ± 8.1		56.6 ± 11.8	
Postop	59.1 ± 11.5	**0.032**	51.7 ± 8.9	**0.029**	53.1 ± 6.4	0.085	56.9 ± 12.4	0.911
Change	5.8 ± 6.5		−6.2 ± 9.3		− 3.2 ± 3.		0.3 ± 2.2	

*IS* Iliac screw, *S2AI* S2-alar-iliac screw, *PI* Pelvic incidence, *Preop* Preoperative, *Postop* Postoperative; change, postoperative - preoperative; Subgroup (C), postoperative change in PI ≥5°; Subgroup (NC), postoperative change in PI < 5°. The *p* value represents the comparison between the preoperative value and postoperative value

### Clinical outcomes and complications

The preoperative back and leg VAS scores and the ODI scores were similar between two groups (Table 4). Although the scores for back and leg, and the ODI scores were greater in IS group than in S2AI group at 3 months postoperatively, the differences between the two groups were not statistically significant. At the last follow-up, the back and leg VAS scores were significantly lower in IS group than in S2AI group (back VAS score: 3.39 ± 1.26 vs 3.92 ± 1.36, *p* = 0.044; leg VAS score: 3.52 ± 1.03 vs 3.95 ± 1.12, *p* = 0.046). The ODI showed no significant difference between two groups at the last follow-up.

**Table 4:** Comparison of VAS and ODI between the IS and S2AI groups

Clinical Outcomes	IS	S2AI	p
VAS (back)			
Preop	7.97 ± 2.81	7.55 ± 3.12	0.474
3 mos postop	3.37 ± 1.41	3.19 ± 1.14	0.515
Last follow-up	3.39 ± 1.2	3.92 ± 1.36	**0.044**
VAS (leg)			
Preop	7.12 ± 1.93	7.31 ± 2.52	0.652
3 mos postop	3.41 ± 2.12	3.32 ± 1.84	0.830
Last follow-up	3.52 ± 1.03	3.95 ± 1.12	**0.046**
ODI			
Preop	49.54 ± 21.25	48.24 ± 19.67	0.760
3 mos postop	18.31 ± 2.96	17.96 ± 1.43	0.525
Last follow-up	21.64 ± 7.63	24.37 ± 5.94	0.068

*IS* Iliac screw, *S2AI* S2-alar-iliac screw, *Preop* Preoperative, *Postop* Postoperative, *VAS* Visual Analog Scale, *ODI* Oswestry Disability Index

Table 5 described postoperative complications. The occurrence of SIJ pain was significantly higher in S2AI group than in IS group (*p* = 0.029). The S2AI group had significantly more pelvic screw fracture than IS group (S2AI 16.1%% vs IS 3.6%; *p* = 0.035). There were four postoperative infections in IS group compared with two in S2AI group (*p* > 0.05). Rates of PJK, pseudarthrosis, wound dehiscence, or screw loosening were similar between two groups.

**Table 5:** Comparison of postoperative complications and reoperations between the IS and S2AI groups

Variable, n (%)	IS (*n* = 111)	S2AI (*n* = 31)	*p*
SIJ pain	10 (9)	8 (25.8)	**0.029**
Pelvic screw loosening	18 (16.2)	4 (12.9)	0.865
Pelvic screw fracture	4 (3.6)	5 (16.1)	**0.035**
Pseudarthrosis	3 (2.7)	2 (6.5)	0.653
Infection (deep)	4 (3.6)	2 (6.5)	0.848
Wound dehiscence	1 (0.9)	1 (3.2)	0.913
Proximal junctional kyphosis	8 (7.2)	4 (12.9)	0.520

*IS* Iliac screw, *S2AI* S2-alar-iliac screw, *SIJ* Sacroiliac joint

## Discussion

**Fig. 3:**
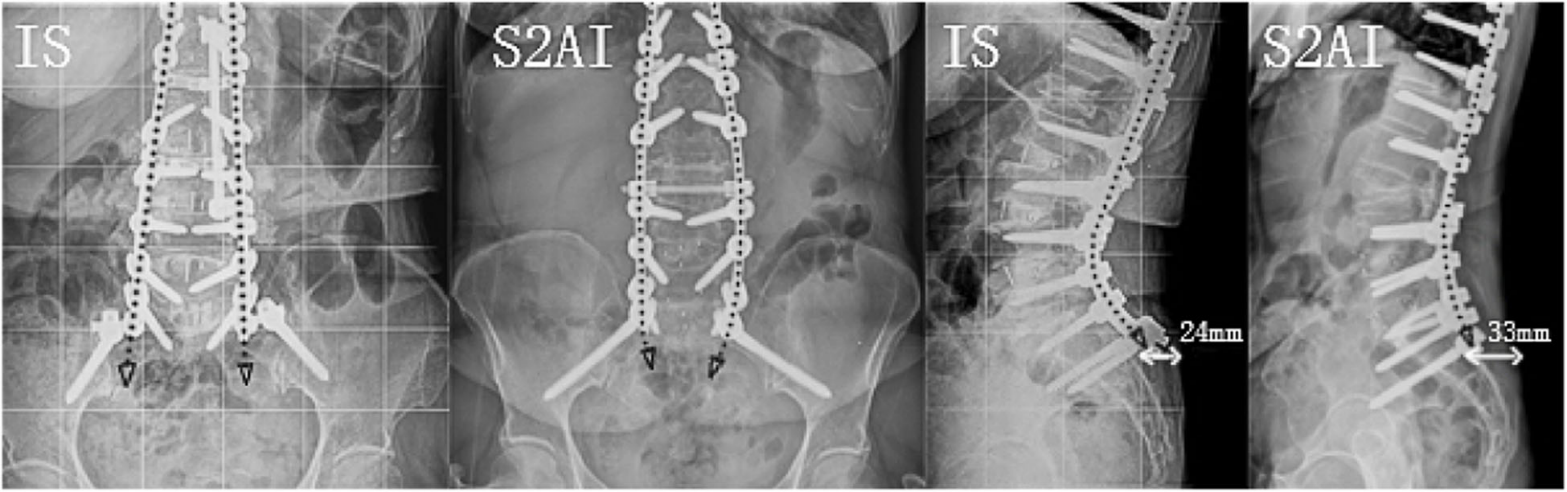
Cantilever force in coronal plane anchoring at ilium between IS and S2AI technique. Rod application in S2AI fixation: curved rod in coronal plane, Low anchoring level in sagittal plane. IS indicates iliac screw; S2AI, S2-alar-iliac screw

The present study demonstrates three major findings:

(1) Historically, both spinopelvic fusion techniques reported in this paper have demonstrated similar effects in terms of stiffness and stability [24, 34]. However, when the differences from baseline to immediately postoperative were compared in the current study, the IS group had significantly greater amounts of correction in LL and ULL. Our study is the first to report that the IS technique could get more correction of lumbar lordosis compared to the S2AI technique used to ASD. There may be two reasons for this: one is that the S2AI technique insertion point is reported to be 15 mm deeper than that in the IS technique [35]. Furthermore, we could observe the curved rod application in the distal area in the coronal plane of S2AI group, which showed worse resistance against distal implant pullout and decreased strain between the screw head and shaft. The S2AI technique is enhanced with iliac buttressing, but the location of the rods is probably too medial to correct more LL. These differences can be further appreciated in Fig. 3 (a graphical rendering of the IS vs S2AI fixation).

(2) PI is a unique anatomical parameter and often considered a constant value after maturity [36]. Most surgeons suggest PI is the key parameter needed to estimate the ideal LL to be restored after spine surgery. However, the current literature [37–39] questions this characteristic of constant PI, claiming that PI may change under certain circumstances. In addition, PI could be changed by motion of the SIJ if it is influenced by various forces due to joint motion or position during supine, sitting, or locomotion behaviors. Thus, the laxity of the SIJ serves as one basis for the change in PI. Nutation increases PI and counternutation decreases PI due to motion at the SIJ [40]. The changes in PI occur in patients who undergo lumbosacral fixation using the IS technique while the changes associated with the S2AI technique have not been well established. Five degrees was determined as a cutoff threshold because deviation may occur owing to varied postures during imaging and measurement and the magnitude of SIJ movement in adulthood has been reported to be 1–4° of rotation [41–43]. Our hypothesis is that mobility in the SIJ could determine the difference in the final shape of the pelvis after surgery, and different surgical techniques would lead to different outcomes in this parameter. The SIJ is six times more resistant to lateral forces than the lumbar spine, and approximately one-half as resistant to axial direction and rotation forces [44]. Hence, stress on the SIJ could increase after spinal fusion that could accelerate degenerative changes, resulting in an increase in motion, particularly that which occurs more frequently after SPF [45, 46]. Specifically, we hypothesize that forced extension of the hips and lumbar spine on the operating table could cause anterior rotation of the sacrum into the pelvis, resulting in an intraoperative increase in PI. If the IS technique was used, this change would be fixed, with a consequent postoperative increase in the observed PI.

Pelvic retroversion generates a reaction force on the SIJ in patients with sagittal malalignment, destabilizing the joint, particularly if combined with degeneration. During surgery, the S2AI screws were placed through the SIJ, reconstructing the morphology of the pelvis. As a consequence, we observed a dramatic decrease in PI. The discrepancy between our postoperative change in PI and that reported by other studies [47] indicates that the potential mechanism may be related to multiple factors. According to cadaveric data [21], over half of S2AI surgeries violated the SIJ, which could potentially affect PI. In addition, postoperative changes in PI are a complicated biological phenomenon in vivo, which are challenging to test in cadavers or biomechanically. However, this result provided some insight into how spinal alignment may be affected by instrumentation and how it is translated into clinical outcomes, though further prospective study is warranted.

(3) The S2AI Group showed a significantly higher rate of pelvic screw breakage than the IS Group. There may be two reasons for this: one is that the acute angle that develops between the screw head and shaft of the screw, may be more prone to failure in the extremes of head-shaft angulation. The second reason may be that the distribution of stresses into two connections with the IS as opposed to a single one with the S2AI are different. Further research is needed to develop a better understanding of these particular types of failure.

In addition, hardware prominence and subsequent pain in the buttocks have been established as complications of the IS technique [1]. In this study, no patients were identified as having screw head prominence that either required revision surgery or resulted in pain, wound dehiscence, or poor cosmesis, which was inconsistent with previously reported results [12, 48]. The difference in lower rate of wound complications associated with the IS technique may be attributed to the fact that we minimized the screw head profile when we deeply recessed pelvic screws into the PSIS specifically to avoid the issue of prominence. Our results suggest that IS fixation is an effective method for SPF. Ishida et al. [49] reported significantly higher rates of symptomatic implant prominence in an IS fixation group. In their study, the screw placement technique was not described and no mention was made of recessing the screw head into the ilium to minimize prominence. Rates of pseudarthrosis at L5-S1 and reoperation due to PJF were similar between their groups.

Limitations of our study include its retrospective design with small sample size and the asymmetry in the number of patients of two groups. However, this is a preliminary study conducted prior to further study in ASD population. Despite these limitations, we believe that the results of this study will be helpful clinically for many spine surgeons.

## Conclusions

Compared with the S2AI technique, the IS technique usable larger cantilever force demonstrated more correction of lumbar lordosis, and possible increase in pelvic incidence. Further study is warranted to clarify the clinical impaction of these results.

## Data Availability

The datasets used and/or analyzed during the current study are available from the corresponding author on reasonable request.

## Abbreviations

ASD: Adult spinal deformity
IS: Iliac screw
S2AI: S2-alar-iliac screw
SPF: Spinopelvic fixation
SVA: Sagittal vertical axis
TK: Thoracic kyphosis
PT: Pelvic tilt
PI: Pelvic incidence
LL: Lumbar lordosis
ULL: Upper lumbar lordosis
LLL: Lower lumbar lordosis
PJK: Proximal junctional kyphosis
UIV: Upper instrumented vertebra
SIJ: Sacroiliac joint
ASA: American Society of Anesthesiologists
EBL: Estimated blood loss
VAS: Visual Analog Scale
ODI: Oswestry Disability Index
